# A Novel Method to Describe Early Offspring Body Mass Index (BMI) Trajectories and to Study Its Determinants

**DOI:** 10.1371/journal.pone.0157766

**Published:** 2016-06-21

**Authors:** Sophie Carles, Marie-Aline Charles, Anne Forhan, Rémy Slama, Barbara Heude, Jérémie Botton, EDEN mother child study group

**Affiliations:** 1 Early determinants of the child’s health and development Team (ORCHAD), INSERM, UMR1153 Epidemiology and Biostatistics Sorbonne Paris Cité Center (CRESS), Paris, F-75014 France; 2 Paris Descartes University, Paris, France; 3 Univ Paris-Sud, Villejuif, France; 4 Team of Environmental Epidemiology applied to Reproduction and Respiratory Health, Inserm, CNRS and University Grenoble Alpes joint research center, Institute of Advanced Biosciences, U1209, Grenoble, France; 5 Grenoble Alpes University, Institute of Advanced Biosciences, U1209, Grenoble, France; 6 Laboratoire de biomathématique, Faculté de Pharmacie, Univ Paris-Sud, Châtenay-Malabry, F-92290, France; INIA, SPAIN

## Abstract

**Background:**

Accurately characterizing children’s body mass index (BMI) trajectories and studying their determinants is a statistical challenge. There is a need to identify early public health measures for obesity prevention. We describe a method that allows studies of the determinants of height, weight and BMI growth up to five years of age. We illustrated this method using maternal smoking during pregnancy as one of the early-life factors that is potentially involved in prenatal programming of obesity.

**Methods:**

Individual height and weight trajectories were fitted using the Jenss-Bayley model on 28,381 and 30,515 measurements, respectively, from 1,666 children to deduce BMI trajectories. We assessed global associations between smoking and growth trajectories and cross-sectional associations at specific ages.

**Results:**

Children exposed in late pregnancy had a 0.24 kg/m^2^ (95% confidence interval: 0.07, 0.41) higher BMI at 5 years of age compared with non-exposed children. Although the BMIs of children exposed during late pregnancy became significantly higher compared with those of non-exposed children from 2 years onwards, the trajectories began to diverge during the first weeks of life.

**Conclusion:**

Our method is relevant for studies on the relationships between individual-level exposures and the dynamics and shapes of BMI growth during childhood, including key features such as instantaneous growth velocities and the age or BMI value at the BMI infancy peak that benefit from the monotonic pattern of height and weight growth.

## Introduction

Relationships between patterns of growth in infancy and childhood and the risk of later chronic diseases such as obesity, type 2 diabetes, cardiovascular diseases have been well described [1–3]. Many studies are now focusing on the determinants of growth. They are mainly studied at one or few time points, but there is a need to analyse their association more globally with longitudinal pattern of growth of children for a better understanding of their consequences. The paucity of longitudinal studies and the lack of relevant methodologies do not allow the precise characterization of the pattern of growth of children and the identification of when the difference in the body mass index (BMI) between different groups emerges.

In a broad context, group- and individual-based approaches are the main methods used to model BMI in childhood. Group-based approaches consider population heterogeneity in trajectories to identify subgroups sharing similar growth patterns [4,5], but the relevance of the number of groups chosen by the analyst can be criticized [6]. Individual-based approaches provide individual BMI trajectories, but the non-monotonic shape of the BMI may require complex modeling methods. Models using (fractional) polynomials [7,8] require the determination of the relevant polynomial order and combination of power(s). High order polynomials better fit the data but often exhibit a poor fit at the extremes [8]. Spline models [9,10] are more flexible, but the number and localization of the knots need to be chosen with some degree of arbitrariness. Moreover, these methods do not provide biologically interpretable parameters for growth.

We describe a method that allows studies of the determinants of an offspring’s height, weight and BMI growth up to five years of age based on non-linear modeling of the individual height and weight growth curves [11]. We chose to illustrate this method with maternal smoking during pregnancy as one of the early-life factors that is potentially involved in the prenatal programming of obesity. Indeed, maternal smoking during pregnancy has been consistently associated with an increased risk of obesity in adulthood [12]. In addition, there is growing evidence that this association appears in childhood, but uncertainties remain regarding the timing and the existence of critical time-windows of exposure during pregnancy. We show how our method (later called “indirect modeling, IM”) provides insights into the timing of the emergence of potential BMI differences in early life.

## Materials and Methods

### Population

EDEN is an ongoing French mother-child cohort established to assess pre- and early postnatal determinants of child health and development [13]. Between 2003 and 2006, 2,002 pregnant women were recruited prior to 24 weeks of gestation defined by the date of the last menstrual period in the Nancy and Poitiers hospitals. Exclusion criteria were multiple pregnancies, personal history of diabetes, illiteracy, and intention to deliver outside the university hospital or to move outside the region within 3 years. Spouses willing to take part were invited to participate; the enrolled pregnant women had to complete a questionnaire and attend a clinical examination where their height was recorded by research midwives. Written informed consent was obtained from the parents for themselves and from both parents for the child after delivery. The study was approved by the ethics committee of Kremlin-Bicêtre and declared to the National Committee for Processed Data and Freedom.

Ninety-five enrolled women left the study due to miscarriages, *in utero* deaths or discontinuation of pregnancy for medical reasons. Among the 1,907 enrolled children, 1,666 had more than one postnatal length/height and weight measurement and complete data on potential confounders; these children were included in the analyses.

### Maternal smoking

During the first visit with the research midwives (between 24 and 28 weeks of gestation), the mothers reported their current smoking status (used to characterize the 2^nd^ trimester status), daily cigarette consumption and smoking habits at the beginning of pregnancy (used for the 1^st^ trimester status). After delivery, similar information for smoking at the end of the 3^rd^ trimester of pregnancy was collected by the research midwives (used for the 3^rd^ trimester status). We investigated differences between “Non-smoking mothers”, “Smokers exclusively in the 1^st^ trimester” and “Smokers in late pregnancy” (i.e., smoking at least during the second and/or third trimesters). Additionally, we calculated the average daily number of cigarettes in the second and third trimesters.

### Anthropometric measurements

The child’s birthweight, daily weights on days 1 to 5 and birth length were extracted from the obstetric records. Length/height and weight postnatal measurements were collected from routine checks via mailed questionnaires and during the study’s clinical visits at 1, 3 and 5 years. Mothers reported lengths/heights and weights recorded by their child’s health practitioners on personal child health records in the 4 preceding months (4-month, 8-month, 1-year questionnaires) or trimesters (2-, 3-, 4- and 5-year questionnaires). At the 1-year visit, mothers were weighed (to the nearest 0.1 kg) alone and holding their child and the child’s weight was determined by subtracting the two measurements. The child’s weight was directly measured at the 3- and 5-year visits. The height was measured to the nearest 0.5 cm.

The median [min-max] numbers of measurements were 18 [2–31] and 19 [2–32] for height and weight, respectively (S1 Table). Among the enrolled children, 1,203 (72%) and 1,208 (73%) had at least one height and weight measurement after four years, respectively.

### Potential confounders

During the first visit, the maternal height was measured to the nearest 0.2 cm by a midwife and the mothers reported their pre-pregnancy weight, age and education level. Their pre-pregnancy BMI was calculated as weight (in kilograms) for measured height (in meters) squared. We determined the overweight (including obesity) status as a BMI ≥25 kg/m^2^. Breast-feeding data were extracted from the medical records during the hospital maternity stay, at discharge and through the mothers’ self-administered postnatal mailed questionnaires at 4 and 8 months and at 1 and 2 years. The duration of any (exclusive or partial) breast-feeding practices was calculated [14] to identify children breast-fed less than 3 months or longer.

### Growth trajectory modeling

Using a recently proposed methodology [11], the individual weight and height trajectories were estimated using the non-linear Jenss-Bayley equation through a mixed effects modeling approach including variance modeling to take heteroscedasticity into account (S1 File). By adding individual random effects in each equation parameter, a set of parameters was used to characterize each child’s growth trajectory. To bypass neonatal weight loss not correctly fitted by the model, birthweight measurements were excluded and the minimal weight in the first four days of life was selected for each child. The models were fitted using the ‘SAEMIX’ package in the R software [15].

Given their non-monotonic shape in early childhood, the BMI trajectories were not directly modeled but were calculated from the modeled height and weight [11]. The mean population and individual BMI trajectories were determined using the height and weight equations (S1 File).

### Statistical analysis

**Fig 1:**
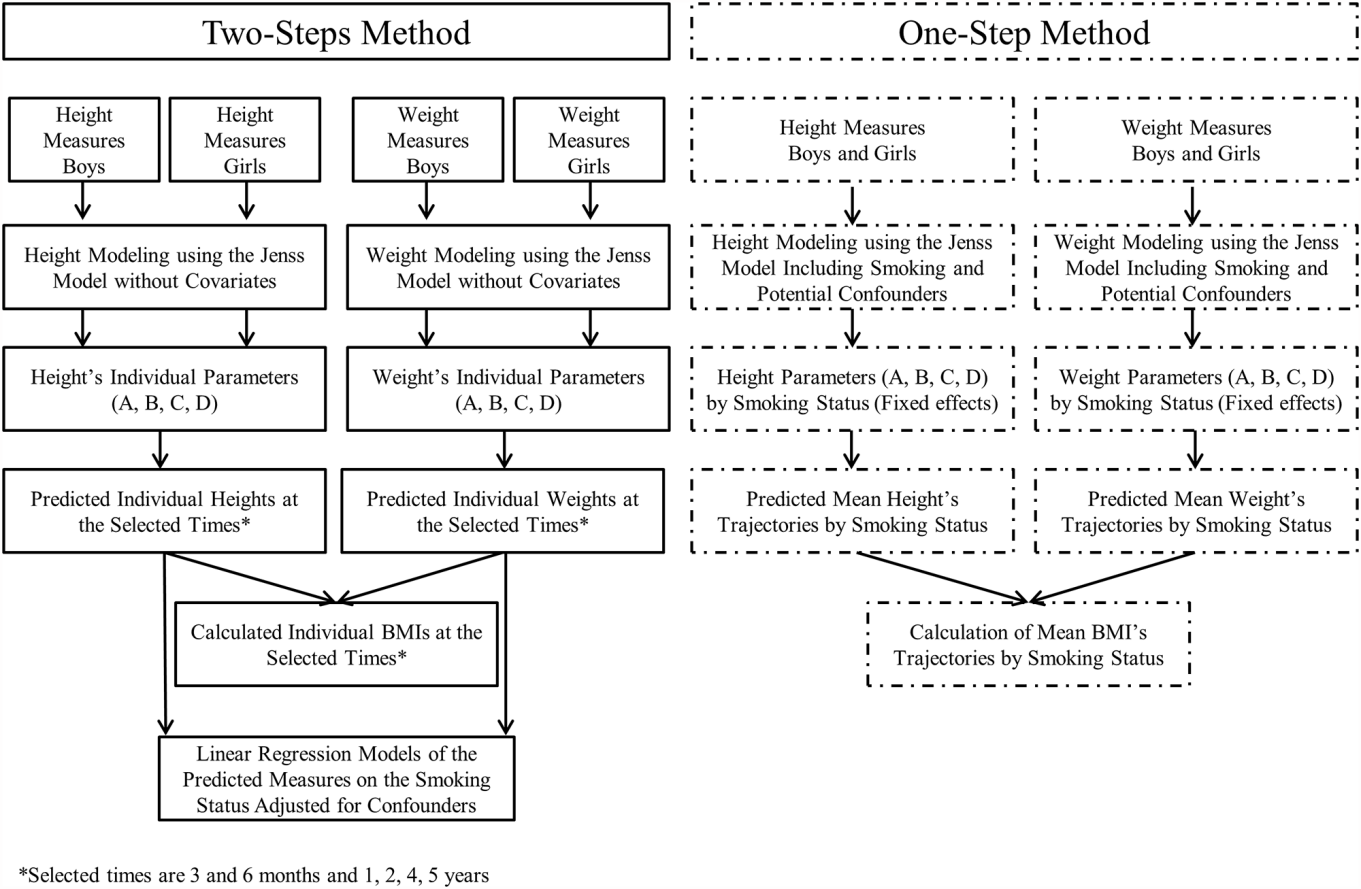
Levels of the “One-step” and “Two-step” Modeling Methods of Growth for Children from the EDEN study aged from birth to 5 years.

Whether the child’s global weight or length/height growth shape differed according to the maternal smoking status was assessed using the “one-step” method. Associations between maternal smoking and anthropometric parameters at birth, the predicted height, weight and BMI, their corresponding velocities at specific ages, and age/BMI at the BMI infancy peak were assessed using the “two-step” method (Fig 1). The analyses were performed on R v3.0 and *P* values <0.05 were considered significant.

#### One-step approach

Gender and potential confounders (maternal education level, age, BMI, breast-feeding duration and recruitment center) were introduced as fixed effects in the length/height and weight models. The general form of the length/height model is given (Eq 1A) and the form decomposing the individual parameters is also detailed (Eq 1B).

$$Height_{i,j}=\text{exp}\left(A_{H_{i}}\right)+\text{exp}\left(B_{H_{i}}\right)*t_{i,j}+\text{exp}\left(C_{H_{i}}\right)*\left(1-\text{exp}\left(-\text{exp}(D_{H_{i}})*t_{i,j}\right)\right)+e_{i,j}$$ (1A)

$$Height_{i,j}=\text{exp}\left(\mu_{A_{H}}+\beta_{A_{H}}*Z+u_{A_{H_{i}}}\right)+\text{exp}\left(\mu_{B_{H}}+\beta_{B_{H}}*Z+u_{B_{H_{i}}}\right)*t_{i,j}+\text{exp}\left(\mu_{C_{H}}+\beta_{C_{H}}*Z+u_{C_{H_{i}}}\right)*\left(1-\text{exp}\left(-\text{exp}(\mu_{D_{H}}+\beta_{D_{H}}*Z+u_{D_{H_{i}}})*t_{i,j}\right)\right)+e_{i,j}$$ (1B)

**Fig 2:**
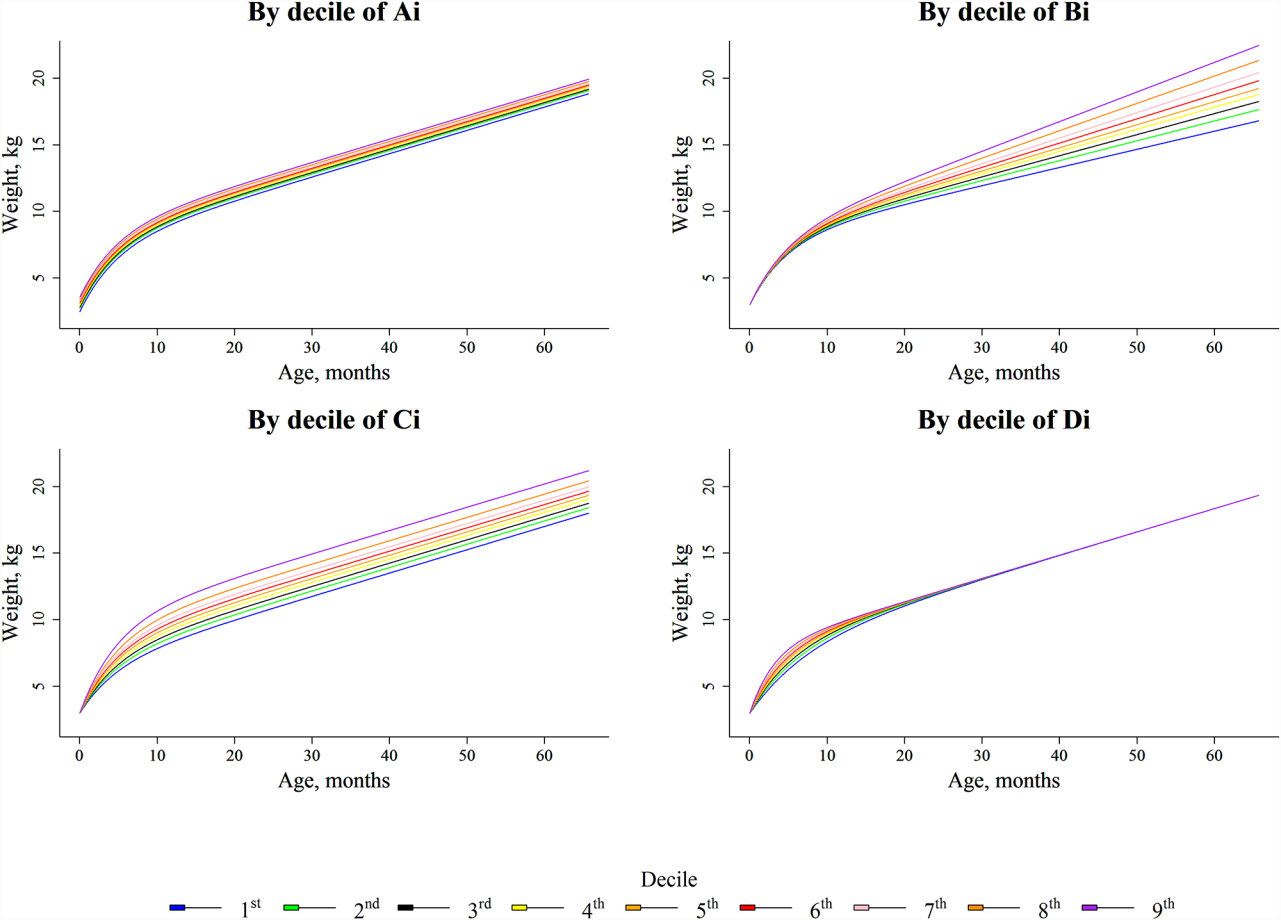
Weight Growth Trajectories from birth to 5 years by deciles of weight growth parameters (Ai, Bi, Ci and Di) in Children of the EDEN Study, France, 2003–2012.

*Height*_*i*,*j*_ describes the height (cm) of child *i* at time *t*_*i*,*j*_ (days), exp(*A*_*H*_) describes the birth length, exp(*B*_*H*_) describes the growth velocity beyond two years, C_H_ is the spurt of growth in the first months of life and *D*_*H*_ is the curvature of the trajectories in the first age period. The parameter definitions are illustrated in Fig 2 and in S1 File (Fig A).

Fig 2 describes the influence of each of the parameters (and more specifically their increase) (Table A in S1 File provides decile values) on the growth of the global shape. $\beta_{A_{H}},\;\beta_{B_{H}},\;\beta_{C_{H}}$, and $\beta_{D_{H}}\beta_{\text{AH}},\beta_{\text{BH}},\beta_{\text{CH}},\beta_{\text{DH}}$ represent covariate effects from vector *Z* (maternal smoking status during pregnancy; potential confounders) on *A*_*H*_, *B*_*H*_, *C*_*H*_, and *D*_*H*_, respectively. $u_{A_{H}},\;u_{B_{H}},u_{C_{H}}$, and $u_{D_{H}}{\text{u}}_{\text{AH}},{\text{u}}_{\text{BH}},{\text{u}}_{\text{CH}},{\text{u}}_{\text{DH}}$ are individual random effects of *A*_*H*_, *B*_*H*_, *C*_*H*,_ and *D*_*H*_ respectively. *e*_*i*,*j*_ is the residual error. $\mu_{A_{H}},\;\mu_{B_{H}},\;\mu_{C_{H}}$, and $\mu_{D_{H}}$ represent the fixed effects of *A*_*H*_, *B*_*H*_, *C*_*H*_, and *D*_*H*_, respectively, for children with *Z* = 0. The weight was modeled similarly. As the first measurement is the minimal weight within the first 4 days, *A*_*W*_ corresponds to the extrapolation of the birth weight without considering neonatal weight loss; thus, the values are lower than the observed measurements. Longitudinal associations between maternal smoking and the length/height and weight trajectories were assessed through comparison of the nested models using likelihood ratio tests.

The BMI of individual *i* at time *j* was predicted as $BMI_{i,j}=\frac{Weight_{i,j}}{{\left(Height_{i,j}/100\right)}^{2}}$ with the weight expressed in kg.

#### Two-step approach

Individual height, weight and BMI were predicted at 0.25, 0.5, 1, 2, 4 and 5 years using individual equations fitted separately for boys and girls without covariates. The individual height (Eq 2), weight and BMI (Eq 3) velocities were calculated at these ages by calculating the first-derivative equations.

$$HeightVelocity_{i,j}=\text{exp}\left(B_{H_{i}}\right)+\text{exp}\left(C_{H_{i}}+D_{H_{i}}-\text{exp}\left(D_{H_{i}}\right)*t_{i,j}\right)$$ (2)

$$BMIVelocity_{i,j}=\frac{WeightVelocity_{i,j}*{\left(\frac{Height_{i,j}}{100}\right)}^{2}-2*\frac{Height_{i,j}}{100}*\frac{HeightVelocity_{i,j}}{100}*Weight_{i,j}}{{\left(\frac{Height_{i,j}}{100}\right)}^{4}}$$ (3)

The individual daily BMI velocities were calculated from birth to five years of age. The age at the BMI infancy peak was the time point when the BMI velocity signs changed (*BMIVelocity*_*i*,*j*_/*BMIVelocity*_*i*,*j*+1_) < 0); the BMI at the BMI infancy peak was calculated for this age. The BMI infancy peaks identified after 2 years of age were excluded from the analyses after the graphical examination of trajectories (n = 49).

Associations between the smoking status and measured birth length/weight, predicted weight, length/height and BMI at the selected ages as well as the age and BMI at the BMI infancy peak were assessed using multivariable linear regression models adjusted for confounders.

### Complementary analysis

To explore the dose-response relationship between maternal smoking in late pregnancy and the child’s growth, the analyses were repeated considering the average number of cigarettes smoked daily in the second and third trimesters as the main exposure.

To evaluate the association between postnatal smoking and growth, we compared the children’s growth from pre- and postnatal non-smoking mothers and non-smoking fathers to the children from non-smoking mothers during pregnancy that (re-)started smoking after birth or whose father smoked postnatally.

### Sensitivity analyses

The analyses were repeated after excluding children born before 37 weeks of gestation to assess any confounding by preterm children. To assess the potential selection bias caused by missing length/height or weight data, the analyses were repeated after excluding children lacking measurements after 4 years of age. To discuss potential differences with other BMI modeling approaches, we replicated the 2-step approach using fractional polynomials and splines. Finally, we report analyses on anthropometric measurements from clinical visits at 1, 3 and 5 years.

## Results

### Population characteristics

Among the enrolled mothers, 24% smoked at least once during pregnancy: 132 (7.9%) exclusively in the first trimester and 275 (16.5%) also during the second and/or third trimester(s) (Table 1). These prevalences were similar for mothers with missing data on confounders (8.5% and 16.5%, respectively).

**Table 1:** Children (n = 1,666) and Parental Characteristics by Maternal Smoking Status during pregnancy from the EDEN study, 2003–2012.

	No smoking (*n* = 1259, 75.57%)		Exclusively the 1^st^ trimester (*n* = 132, 7.92%)			Late pregnancy (*n* = 275, 16.51%)		
	%	Mean (SD)	%	Mean (SD)	*P* Value^a^	%	Mean (SD)	*P* Value^b^
Maternal Characteristics								
Age		30.1 (4.6)		28.5 (5)	<0.001		28.5 (5.4)	<0.001
Height, cm		163.6 (6.1)		163.7 (6)	0.85		163.3 (6.4)	0.54
Weight, kg		62.5 (12.7)		60.5 (12.1)	0.08		61.6 (12.7)	0.33
BMI^c^, kg/m^2^		22.2 (14.9–60.4)		21.4 (15.6–38)	0.02		21.8 (14–43)	0.28
Overweight or Obese	25.9		23.5		0.09	26.6		0.08
Center (Poitiers)	45.9		50.8		0.29	53.1		0.03
Education level								
High school or less	38.8		51.5		<0.001	69.8		<0.001
2-year university degree	23.8		20.4			15.6		
3-year university degree	37.4		28			14.6		
Primiparity	43.8		56.8		0.004	43.3		0.86
Number of cigarettes per day								
1^st^ Trimester				6.0 (1–35)			8.0 (1–40)	
2^nd^ Trimester							5.0 (1–20)	
3^rd^ Trimester							5.0 (1–25)	
Paternal Characteristics								
BMI, kg/m^2^		24.8 (15.8–40.8)		24.5 (18–35.2)	0.12		24.3 (17.6–35.5)	0.07
Smoking status	29.7		62.1		<0.001	75.1		<0.001
Child Characteristics								
Male Sex	52.3		49.2		0.51	51.6		0.85
Gestational age, weeks^c^		40 (28–42)		40 (34–42)	0.92		39 (30–42)	0.36
Birth length, cm		49.71 (2.31)		49.43 (2.02)	0.19		49.03 (2.39)	<0.001
Birth weight, g		3311 (507)		3316 (455)	0.92		3157 (486)	<0.001
Breast feeding Yes/No	76.9		71.2		0.14	61.8		<0.001
Breast feeding ≥3 months	50.2		40.9		0.04	26.9		<0.001
Breast-feeding duration in breast-fed child, months		4 (0.2–13)		3 (0.2–13)	<0.001		2 (0.2–13)	<0.001
Prematurity	5.8		2.3		0.09	4.4		0.35

Abbreviations: BMI, body mass index; SD, standard deviation

^a, b^Comparison between characteristics of mothers who smoked exclusively during the 1^st^ trimester, their children and partners and those of non-smokers using the Χ^2^, *t* tests or Man-Whitney-Wilcoxon tests as appropriate

^c^Median (min-max) presented due to left or right skew

Maternal smoking was inversely associated with the age at delivery, education level and breast feeding initiation and duration. Smokers in late pregnancy delivered neonates with smaller birth lengths and weights than non-smokers (Table 1).

### Growth

The ‘one-step’ method assesses associations between maternal smoking and length/height or weight trajectories as a whole and the effects on the four-specific Jenss-Bayley model parameters for length/height and weight (Table 2).

**Table 2:** Mean differences in Height and Weight Model Parameters in each Maternal smoking category and the reference category (No smoking) using the (One-step Method)^a^ in Children aged 0–5 years in the EDEN Study, 2003–2012.

Outcomes	A		B		C		D		Global *P*value
	Birth length		Growth Velocity		Spurt of Growth		Curvature Degree		
	Extrapolation of Birth weight		-Childhood-		-First Months-		-First Months-		
	β_A_	CI 95%	β_B_	CI 95%	β_C_	CI 95%	β_D_	CI 95%	
Height									<0.001
Exclusively the 1^st^ trimester	-0.003	[-0.012,0.005]	0.028	[-0.001,0.057]	-0.022	[-0.057,0.013]	0.074*	[0.010,0.137]	
Late pregnancy	-0.015*	[-0.021,-0.008]	0.022	[-0.001,0.046]	0.004	[-0.024,0.031]	0.038	[-0.011,0.087]	
Weight									0.03
Exclusively the 1^st^ trimester	0.008	[-0.025,0.041]	-0.004	[-0.050,0.042]	0.016	[-0.036,0.068]	-0.023	[-0.099,0.054]	
Late pregnancy	-0.037*	[-0.062,-0.012]	0.035	[-0.001,0.071]	0.008	[-0.032,0.048]	0.022	[-0.037,0.081]	

Abbreviations: BMI, body mass index, CI, confidence interval.

^a^Adjusted for maternal education level, BMI and age at delivery; breast-feeding duration; recruitment center; child's gender.

*P<0.05.

The ‘two-step’ method shows associations between maternal smoking and predicted height, weight, and BMI and their corresponding velocities at specific ages (Table 3).

**Table 3:** Mean Differences in Predicted Anthropometric Outcomes from Birth to 5 Years in each Maternal Smoking Category and the Reference Category (No Smoking)^a^ (Two-step method).

Outcomes by maternal smoking status	Birth		3 months		6 months		1 year		2 years		4 years		5 years	
	β	95% CI	β	95% CI	β	95% CI	β	95% CI	β	95% CI	β	95% CI	β	95% CI
Length/Height, cm														
Exclusively the 1^st^ trimester	-0.24	[-0.63,0.15]	0.16	[-0.22,0.53]	0.23	[-0.16,0.62]	0.18	[-0.24,0.60]	0.06	[-0.45,0.57]	0.20	[-0.46,0.87]	0.35	[-0.40,1.11]
Late pregnancy	-0.63*	[-0.92,-0.33]	-0.40*	[-0.68,-0.12]	-0.30*	[-0.59,0.00]	-0.23	[-0.55,0.08]	-0.19	[-0.57,0.19]	-0.06	[-0.56,0.44]	0.02	[-0.55,0.58]
Weight, g														
Exclusively the 1^st^ trimester	28	[–61,117]	15	[–98,129]	39	[–98,176]	75	[–95,245]	101	[–119,321]	125	[–206,455]	137	[–261,535]
Late pregnancy	-120*	[–187,–54]	-53	[–138,33]	-22	[–125,81]	33	[–95,161]	121	[–44,287]	249*	[1,498]	307*	[8,606]
BMI, kg/m^2^														
Exclusively the 1^st^ trimester	0.25	[-0.01,0.52]	-0.05	[-0.27,0.17]	-0.04	[-0.27,0.19]	0.03	[-0.19,0.26]	0.10	[-0.12,0.31]	0.04	[-0.17,0.25]	0.00	[-0.22,0.22]
Late pregnancy	-0.16	[-0.37,0.04]	0.08	[-0.09,0.24]	0.11	[-0.07,0.28]	0.16	[-0.01,0.33]	0.23*	[0.07,0.39]	0.25*	[0.09,0.41]	0.24*	[0.07,0.41]
Height velocities, cm/month														
Exclusively the 1^st^ trimester			0.049*	[0.002,0.096]	0.007	[-0.023,0.036]	-0.015	[-0.039,0.009]	-0.003	[-0.015,0.009]	0.011	[0.000,0.023]	0.013*	[0.001,0.025]
Late pregnancy			0.049*	[0.013,0.084]	0.022*	[0.000,0.044]	0.005	[-0.013,0.022]	0.004	[-0.005,0.013]	0.006	[-0.002,0.015]	0.006	[-0.003,0.015]
Weight velocities, kg/month														
Exclusively the 1^st^ trimester			0.007	[-0.012,0.026]	0.008	[-0.006,0.022]	0.004	[-0.005,0.013]	0.001	[-0.005,0.008]	0.001	[-0.006,0.008]	0.001	[-0.006,0.008]
Late pregnancy			0.011	[-0.003,0.026]	0.010	[-0.001,0.020]	0.009*	[0.002,0.016]	0.006*	[0.001,0.011]	0.005	[0,0.01]	0.005	[0.000,0.010]
BMI velocities, kg/m^2^/month														
Exclusively the 1^st^ trimester			-0.068*	[-0.135,-0.001]	0.011	[-0.012,0.035]	0.011	[-0.001,0.023]	0.001	[-0.005,0.006]	-0.003	[-0.007,0.001]	-0.003	[-0.007,0.000]
Late pregnancy			0.023	[-0.028,0.073]	0.009	[-0.009,0.026]	0.009	[0.000,0.017]	0.003	[-0.001,0.008]	0.000	[-0.003,0.003]	-0.001	[-0.003,0.002]
Age at BMI peak^b^, days														
Exclusively the 1^st^ trimester					7.31	[-11.93,26.55]								
Late pregnancy					4.97	[-9.46,19.39]								
BMI at BMI peak^b^, kg/m^2^														
Exclusively the 1^st^ trimester					-0.04	[-0.27,0.19]								
Late pregnancy					0.12	[-0.05,0.29]								

Abbreviations: BMI, body mass index, CI, confidence interval.

^a^Adjusted for maternal education level, BMI and age at delivery, breast-feeding duration, recruitment center, and child's gender.

^b^Analyses restricted to subjects with valid data for age and BMI at the BMI peak (identifiable BMI peak and BMI peak before 732 days). The number of children available for the analysis was 1,617.

*P<0.05.

The overall fits of the models were good (Figs A and B in S2 File). Although the fit was good for weight because no trend was observed among the residuals across time, the model fit the data somewhat less adequately for height. However, the distribution of the BMI residuals over time and the individual fitted trajectories illustrated the good ability of the indirect modeling to fit the BMI data (Figs A and C in S2 File).

#### Length/height growth

**Fig 3:**
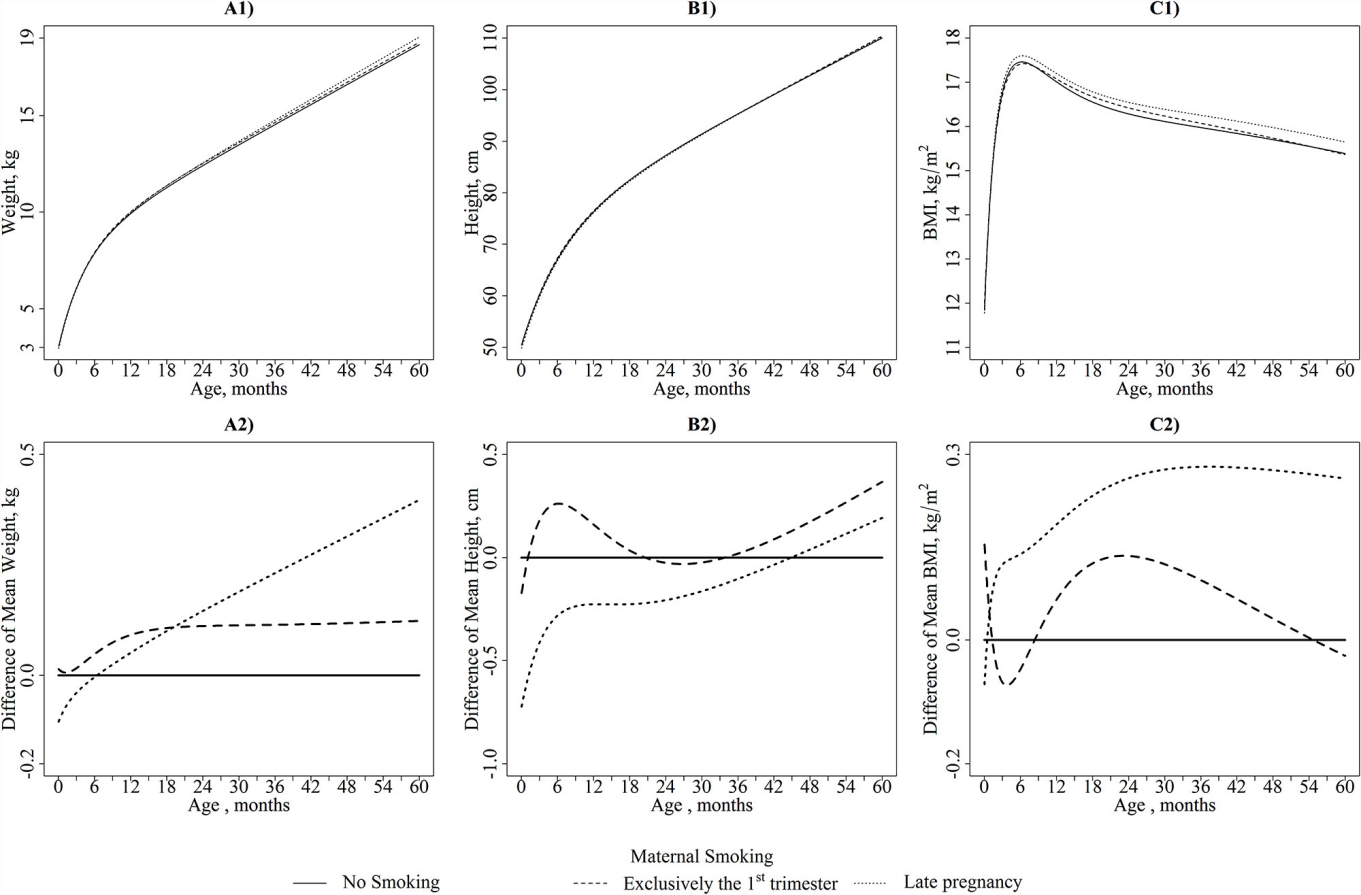
A1) Weight (kg), B1) Height (cm), and C1) BMI (kg/m^2^): Mean Trajectories in each category of Maternal Smoking for children from the EDEN Study aged from birth to 5 years. A2) Weight (kg), B2) Height (cm), and C2) BMI (kg/m^2^): Differences in Trajectories between each category of Maternal Smoking and the Reference Category (No Smoking) for children from the EDEN Study aged from birth to 5 years. Mean Trajectories are illustrated for Boys breastfed less than 3 months with 30 year-old non-overweight mothers with a high-school education or less born in Poitiers.

Fig 3B1 illustrates the height growth curve by smoking categories for children with given socio-demographic characteristics, and Fig 3B2 shows the differences in the mean height trajectories between children from non-smoking mothers and children from mothers who smoked exclusively during the 1^st^ trimester or until late pregnancy.

The child length/height trajectories differed among children exposed to maternal smoking during pregnancy and children from non-smokers (p<0.001).

Compared with no maternal smoking, smoking during the 1^st^ trimester exclusively was associated with lower C and significantly higher D parameters. This finding illustrates a faster early length growth for a short period of time (up to 3 months). These children also exhibited faster height growth velocities at 5 years compared to the children from non-smokers, although their height was not significantly greater at these ages (Table 3).

Compared with no maternal smoking, maternal smoking during late pregnancy was associated with a 0.015 lower A parameter (95% CI: -0.021, -0.008). This difference represented a 0.63 cm shorter length at birth in children whose mothers smoked during late pregnancy compared to children born to non-smoker mothers.

Compared to children from non-smokers, children whose mothers smoked during late pregnancy had a lower average length from birth to 6 months. They exhibited a significantly faster length growth velocity immediately after birth (higher D parameter compared to children from non-smokers) up to 6 months (Table 3). These children seemed to catch up to the heights of children from non-smokers only at the end of the study period (Table 3).

#### Weight growth

Similar to height, Fig 3A1 illustrates the weight growth curve by smoking categories, and Fig 3A2 shows the differences in the mean weight trajectories between children from non-smoking mothers and children from mothers who smoked exclusively during the 1^st^ trimester or until late pregnancy.

Maternal smoking during pregnancy was significantly associated with a change in the global weight trajectory (p = 0.03).

The average weight trajectory of children whose mothers smoked during the 1^st^ trimester exclusively was similar to that of children from non-smokers (Table 2).

However, children born to mothers who smoked during late pregnancy had weight trajectories that differed from the children of non-smokers. These children had birthweights that were 120 grams lower and tended to have a higher D parameter that was in line with faster growth in the beginning of life. Using the two-step approach, we found that their weight growth was significantly higher at one and two years (0.009 and 0.006 kg/month, 95% CI: 0.002, 0.016 and 0.001, 0.011, respectively) and higher at 4 and 5 years without reaching statistical significance. The higher B parameter in this subgroup was in line with this higher weight growth during childhood. Thus, they surpassed the weight of unexposed children at approximately 2 years and continued to gain more weight thereafter. At ages 4 and 5 years, they had a 249 gram (95% CI: 1, 498) and 307 gram (95% CI: 8, 606) higher mean weight, respectively, compared to children born to non-smokers.

#### BMI growth

Fig 3C1 illustrates the resulting BMI growth curve by smoking categories, and Fig 3C2 shows the differences in the mean BMI trajectories between children from non-smoking mothers and children from mothers who smoked exclusively during the 1^st^ trimester or until late pregnancy.

Children born to non-smokers and to mothers who smoked during the 1^st^ trimester exclusively had quite similar mean BMI trajectories. Smoking during the 1^st^ trimester exclusively was not associated with any age-predicted BMI. The mean BMI trajectory of children from smokers in late pregnancy surpassed the trajectory of the unexposed children in the first couple of months. Although their ages at the BMI infancy peak were comparable, maternal smoking in late pregnancy was associated with a non-significant BMI increase at the BMI infancy peak (0.12, 95% CI: -0.05, 0.29). At 2 years of age, these children had a significantly higher BMI (0.23 kg/m^2^). This difference reached 0.24 kg/m^2^ at 5 years.

### Dose response

A dose-response relationship between maternal smoking in late pregnancy and growth characteristics (Tables A and B in S3 File) was observed. The height and weight A parameters were significantly negatively associated and height D was positively associated with the number of cigarettes smoked per day. A five cigarette per day increase was associated with a 0.39 cm (95% CI: -0.58, -0.20) and 0.21 cm (95% CI: -0.40, -0.02) lower length at birth and at 6 months, respectively, a 82 gram lower measured birthweight, and a 155 gram (95% CI: -1, 312) and 188 gram (95% CI: 0, 376) higher predicted weight at 4 and 5 years, respectively. These results were in line with a 0.018 higher B parameter (95% CI: -0.005, 0.042).

### Sensitivity analysis (S4 File)

The exclusion of the 88 preterm children (Table A in S4 File) and the restriction of the analysis to 1,211 children with data beyond 4 years of age did not change the results. The weights, heights and BMIs of children exposed only postnatally were comparable to these measurements from the non-exposed children (Table B in S4 File). Associations between the predicted BMIs at different time points and maternal smoking status gave close results regardless of the modeling method used (Table C in S4 File). For the subsample of children attending the clinical visits at 1, 3 and 5 years, the associations with maternal smoking status were comparable for the measurements from clinical visits or the model-predicted data (Table D in S4 File).

## Discussion

Our approach provides mutually complementary methods to accurately characterize the determinants of growth in children from birth to five years. For the example of maternal smoking during pregnancy, we showed that although the BMI of children exposed during late pregnancy became significantly higher from 2 years of age onwards compared to unexposed children, their BMI trajectories started to diverge very early in infancy. This finding was explained by a predominant catch-up growth in weight compared to length and reinforced by a faster weight gain from 1 year onwards.

The ‘one-step’ method allows the examination of growth trajectories longitudinally and provides a global test for the relationship between the exposure and weight/height trajectories overall and for period-specific characteristics of growth using the Jenss-Bayley model parameters. The ‘two-step’ method provides statistical tests at specific ages (especially for BMI), thereby providing information on the magnitude of the between-subgroups differences.

The results regarding the associations between maternal smoking and BMI across childhood were very close using our indirect method or the fractional polynomials and splines However, the monotonic pattern of weight and height (in contrast to BMI growth) can be beneficial using our indirect BMI modeling. Our indirect modeling approach also provides additional assets. First, the height and weight model parameters have biological interpretability. Second, the model is less sensitive to outliers compared to non-structural models [16]. Third, our method does not need to collect weight and height measurements at the same time (in comparison with direct modeling), which increases the number of available measurements for modeling. Compared to the study of associations using observed data, our approach takes into account missing data and limits potential selection bias (Table D in S4 File). Furthermore, in contrast to model-predicted data, analyses using the observed data did not allow the assessment of the association at many time points and consequently the accurate characterization of the evolution of differences between the smoking subgroups over time. Finally, the observed data did not allow the assessment of associations with key growth milestones, such as instantaneous growth velocities and age and BMI at the BMI infancy peak.

In terms of weaknesses, the indirect BMI modeling did not allow the assessment of the global association between exposure and BMI trajectories, which can be performed for the height and weight trajectories separately; however, there can be some potential convergence issues when the covariate numbers increase. Additionally, the smoking status at the beginning of pregnancy was taken as a proxy measurement for the 1^st^ trimester status. A high percentage of women quit very early in pregnancy, which could explain the lack of association with birth outcomes in children exposed exclusively during the 1^st^ trimester. Furthermore, assessing the smoking status using a self-reported questionnaire could lead to misclassification bias. We do not expect that the estimated differences in anthropometric measurements between exposed and non-exposed children would be overestimated by underreporting.

In recent reviews of early determinants of overweight or obesity, maternal smoking during pregnancy was consistently associated with adult obesity [12] and child overweight and obesity [17,18]. These reviews were in favor of a long-lasting effect of maternal smoking on health. Our study adds to the growing body of evidence suggesting the emergence of these associations in infancy. The few studies investigating early growth explained the increased BMI by a weight deficit at birth followed by a rapid weight catch-up growth prior to 1 year that lead to a similar or higher weight compared to non-exposed children later in childhood [19–21]. Although exposed children tend to catch up in weight, a persistently shorter height in late childhood was observed [20–22]. Our results on the growth velocities and C and D parameters showed a greater impact of maternal smoking on length than weight growth during the first 6 months of life. Because weight depends on length growth, this discrepancy is in favor of a differential effect on the different compartments of body weight. The BMI trajectory in children exposed during late pregnancy suggests a faster fat mass accretion in the first months of life with a higher BMI at the BMI infancy peak that is maintained throughout childhood.

In our population, a smaller weight and length at birth were associated with maternal smoking during late pregnancy but not with smoking during the 1^st^ trimester exclusively. These results corroborate the association between maternal smoking and impaired fetal growth reported in previous works [23,24]. Furthermore, maternal smoking exclusively during the 1^st^ trimester was not associated with any age-predicted BMI, supporting a study that found no association between maternal smoking in the 1^st^ trimester and the BMI of children up to 4 years [20] but contrasting with another study where a higher risk of being overweight was found in children aged 5 to 7 years [25].

A direct effect of smoking during pregnancy has been postulated to occur through increased maternal and fetal carboxyhemoglobin blood levels, which are responsible for fetal hypoxia and growth retardation [26]. Subsequent associations with postnatal growth might be explained by a deregulation of the hypothalamic centers that regulate food intake, growth and adipocyte numbers [27]. Tobacco might also have a potentiating effect on hypothalamic structures, resulting in impaired insulin signaling and metabolism [27].

The persistent positive association between maternal smoking and overweight children in within-sibling analyses [28] and the higher effect estimates for maternal smoking compared with paternal smoking [29] support intrauterine effects, whereas the attenuated associations between maternal smoking and obesity in young adults in within-family analyses [30] backed by studies with similar [22,31–33] or stronger [34] associations between paternal and maternal smoking statuses and the offspring’s overweight BMI suggest residual confounding by unmeasured shared familial factors. We did not control for lifestyle characteristics. Although an effect of shared familial factors cannot be excluded, we propose that this effect is less likely because the differences appeared very early in life. The fast increase in the BMI during the first weeks of life could suggest that cessation of exposure to tobacco products (e.g., nicotine) immediately after birth may contribute to the observed association as previously speculated [35].

Our findings are consistent with an association between smoking in late pregnancy and upward BMI trajectories as early as the first months of life, resulting in a difference in the BMI that is maintained throughout the infancy and childhood periods. This result supports the concept that infant programming related to fetal exposure to adverse conditions has lasting or lifelong effects. The second and third trimesters of pregnancy could be more critical periods for the induction of lifelong susceptibility to obesity. Our approach based on height and weight modeling offers a convenient method to estimate key trajectory milestones and associations with early-life determinants.

## Supporting Information

S1 File Indirect Body Mass Index Modeling. (DOCX)

S2 File Model Adequacies. (DOCX)

S3 File Dose Effect Analysis. (DOCX)

S4 File Sensitivity Analyses. (DOCX)

S1 Table Number of Measurements available for Modeling the Height and Weight growth of children (0–5 years) from the EDEN study, France, 2003–2012. (DOCX)

## Abbreviations

CI: confidence interval
BMI: body mass index
EDEN: Etude des Déterminants pré et postnatals précoces du développement et de la santé de l’Enfant
