# Supplementary material for: A Novel Method to Describe Early Offspring Body Mass Index (BMI) Trajectories and to Study Its Determinants
Source: PLoS One. 2016 Jun 21;11(6):e0157766. doi: 10.1371/journal.pone.0157766 (PMC4915665; doi:10.1371/journal.pone.0157766)
Supplement: S1 File — (DOCX) [file pone.0157766.s001.docx]

**S1 File.** Indirect BMI modeling

Expected height (cm) or weight (kg), $Y_{i,j}$ at measurement time $t_{i,j}$ (days) for the child $i$ were expressed as follows:

$$\left( \begin{matrix} A_{i} \\ B_{i} \\ C_{i} \\ D_{i} \end{matrix} \right)=\left( \begin{matrix} A \\ B \\ C \\ D \end{matrix} \right)+\left( \begin{matrix} u_{A_{i}} \\ u_{B_{i}} \\ u_{C_{i}} \\ u_{D_{i}} \end{matrix} \right)$$

$$\boldsymbol{\phi}_{i}={\boldsymbol{\phi} + \boldsymbol{u}}_{i}$$

Equation 1.

$$Y_{i,j}=\exp\left( A_{i} \right)+\exp\left( B_{i} \right){*t}_{i,j}+\exp\left( C_{i} \right)*\left( 1-\exp\left( -exp(D_{i}{)*t}_{i,j} \right) \right){+e}_{i,j}$$

The vector of fixed effects $\boldsymbol{\phi}$, is the average value of the individual parameters $\boldsymbol{\phi}_{i}$ of the children included.

The vector of random effects $\boldsymbol{u}_{i}$ represents the deviation of $\boldsymbol{\phi}_{i}$ from their population average ($\boldsymbol{\phi}$) corresponding to between-individual variability, and $e_{i,j}$ are the residuals corresponding to within-individual variability. A combined error model was used to describe the residual error of height and weight. The general form of $Y_{i,j}$ can be expressed as

$$Y_{i,j}={f(\boldsymbol{x}_{i,j},\boldsymbol{\phi}}_{i})+{g(\boldsymbol{x}_{i,j},\boldsymbol{\phi}}_{i},\xi)\varepsilon_{i,j}$$

where $\boldsymbol{x}_{i,j}$ are the regression variables or design variables and $\varepsilon_{i,j}$ are the residual errors. The residual error model is defined by the function $g$ and the parameters $\xi$. The combined error model assumes that $g=a+bf$ and $\xi=(a, b)$.

Random effects have to be normally distributed with mean **0** and a symmetric variance-covariance matrix denoted $\boldsymbol{\Omega}_{\boldsymbol{u}}$, as are the residuals.

$\boldsymbol{u}_{i}\sim N(\boldsymbol{0},\boldsymbol{\Omega}_{\boldsymbol{u}})$

For a better interpretation of the parameters, Fig A illustrates the influence of each of them on the global growth shape. Weight trajectories 1--4 were fitted, respectively, for each decile of A_i_, B_i_, C_i_, and D_i_ (Table A) while fixing the other 3 parameters at the value of the population average. For illustration purposes, independency of parameters was assumed which is more straightforward although not realistic. In reality, the non-null covariance between parameters can result in a larger panel of shapes.

**Table A.** Distributions of Individual Weight Growth Model among Boys and Girls (0-5 years) of the EDEN study, France, 2003-2012

| Parameter | Deciles | | | | | | | | | Mean |
| --- | --- | --- | --- | --- | --- | --- | --- | --- | --- | --- |
|  | 1^st^ | 2^nd^ | 3^rd^ | 4^th^ | 5^th^ | 6^th^ | 7^th^ | 8^th^ | 9^th^ |  |
| A_Wi_ Birth length Extrapolation of birth weight | 0.89 | 0.98 | 1.02 | 1.06 | 1.10 | 1.13 | 1.17 | 1.21 | 1.26 | 1.08 |
|  |  |  |  |  |  |  |  |  |  |  |
| Bw_i_ Growth Velocity -Childhood- | -5.41 | -5.32 | -5.26 | -5.21 | -5.17 | -5.12 | -5.07 | -5.00 | -4.92 | -5.16 |
|  |  |  |  |  |  |  |  |  |  |  |
| Cw_i_ Spurt of Growth -First Months- | 1.27 | 1.38 | 1.46 | 1.53 | 1.59 | 1.65 | 1.71 | 1.79 | 1.91 | 1.59 |
|  |  |  |  |  |  |  |  |  |  |  |
| Dw_i_ Curvature Degree -First Months- | -5.41 | -5.25 | -5.12 | -5.04 | -4.96 | -4.87 | -4.80 | -4.70 | -4.54 | -4.97 |


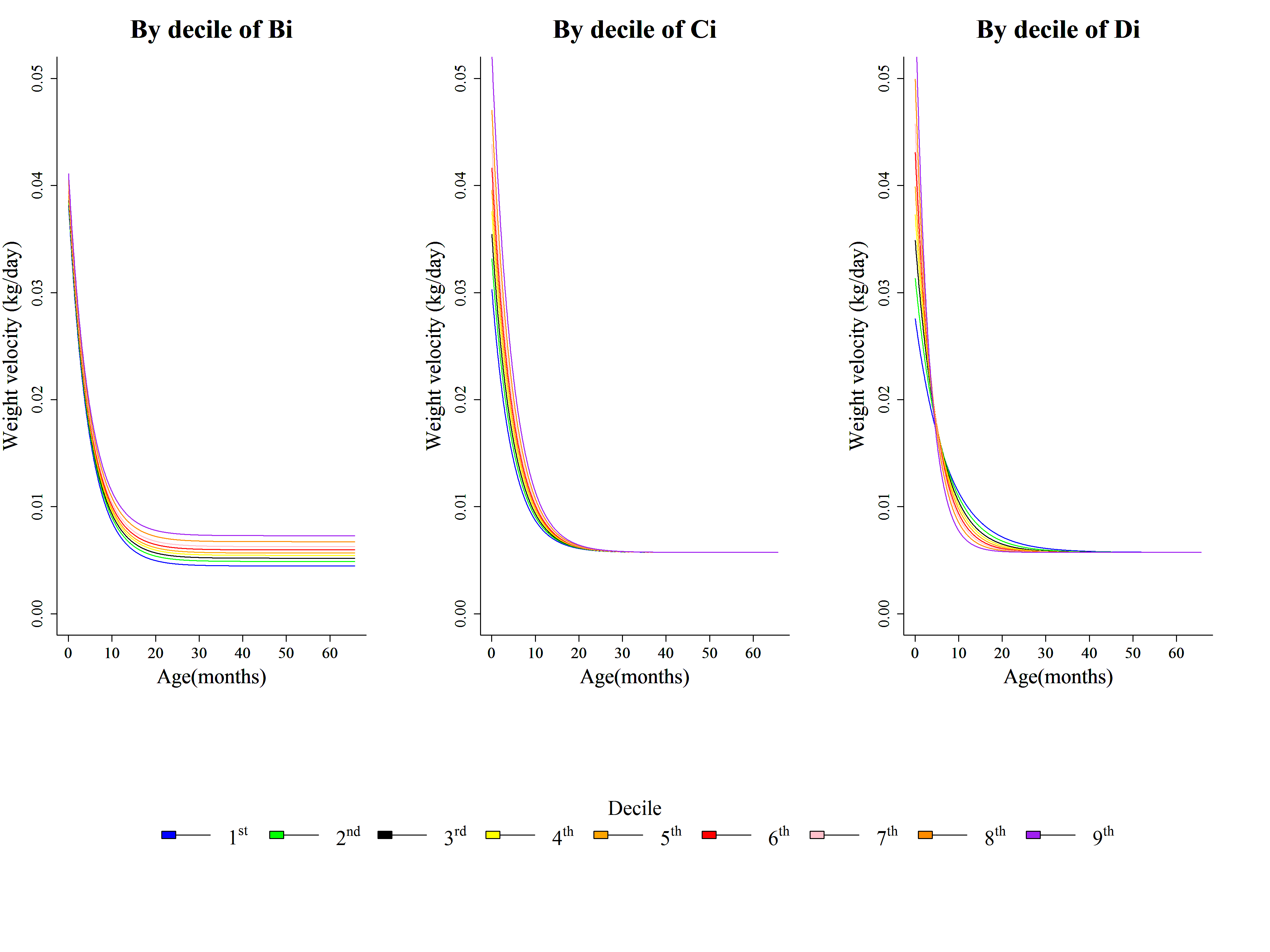


**Fig A.** Weight Growth Velocities from birth to 5 years by decile of weight growth parameters (A_i_, B_i_, C_i_ and D_i_) in Children of the EDEN Study, France, 2003-2012

The A component describes the length at birth or the approximation of the minimal weight in the first days of life. The B component describes the asymptote for the older ages or growth velocity beyond two years. The C component describes the spurt of growth in the first months of life, the difference between birth measure and the value of the later ages asymptote in the Y-axis. The D component describes the curvature in the first period of growth.

The distributions of individual height and weight growth parameters obtained through Jenss modeling in our sample are detailed in the Table B.

**Table B.** Distributions of Individual height and weight growth model parameters in children (0-5 years) of the EDEN study, France, 2003-2012

| Model Parameters Mean (SD) |  | Gender | |
| --- | --- | --- | --- |
|  |  | Boys | Girls |
| Height |  |  |  |
| A_H_ |  | 3.92 (0.04) | 3.90 (0.04) |
| B_H_ |  | -3.89 (0.10) | -3.90 (0.12) |
| C_H_ |  | 3.14 (0.13) | 3.11 (0.15) |
| D_H_ |  | -5.37 (0.26) | -5.45 (0.28) |
| Weight |  |  |  |
| A_W_ |  | 1.10 (0.17) | 1.06 (0.18) |
| B_W_ |  | -5.16 (0.20) | -5.16 (0.21) |
| C_W_ |  | 1.64 (0.23) | 1.52 (0.27) |
| D_W_ |  | -4.94 (0.34) | -5.00 (0.35) |
|  |  |  |  |
| Abbreviation: SD, standard deviation | | | |

Body Mass Index trajectories were deduced using height and weight equations.

**Equation 3.**

$${Weight}_{i,j}=\exp\left( A_{W_{i}} \right)+\exp\left( B_{W_{i}} \right){*t}_{i,j}+\exp\left( C_{W_{i}} \right)*\left( 1-\exp\left( -exp(D_{W_{i}}{)*t}_{i,j} \right) \right){+e}_{i,j}$$

**Equation 4.**

$${Height}_{i,j}=\exp\left( A_{H_{i}} \right)+\exp\left( B_{H_{i}} \right){*t}_{i,j}+\exp\left( C_{H_{i}} \right)*\left( 1-\exp\left( -exp(D_{H_{i}}{)*t}_{i,j} \right) \right){+e}_{i,j}$$

$\left( \begin{matrix} A_{H_{i}} \\ B_{H_{i}} \\ C_{H_{i}} \\ D_{H_{i}} \end{matrix} \right)=\left( \begin{matrix} A_{H} \\ B_{H} \\ C_{H} \\ D_{H} \end{matrix} \right)+\left( \begin{matrix} u_{A_{H_{i}}} \\ u_{B_{H_{i}}} \\ u_{C_{H_{i}}} \\ u_{D_{H_{i}}} \end{matrix} \right)$ and $\left( \begin{matrix} A_{W_{i}} \\ B_{W_{i}} \\ C_{W_{i}} \\ D_{W_{i}} \end{matrix} \right)=\left( \begin{matrix} A_{W} \\ B_{W} \\ C_{W} \\ D_{W} \end{matrix} \right)+\left( \begin{matrix} u_{A_{W_{i}}} \\ u_{B_{W_{i}}} \\ u_{C_{W_{i}}} \\ u_{D_{W_{i}}} \end{matrix} \right)$

With $\left\{ A_{H_{i}}, B_{H_{i}}, C_{H_{i}},D_{H_{i}} \right\}$ and $\left\{ A_{W_{i}}, B_{W_{i}}, C_{W_{i}},D_{W_{i}} \right\}$ the individual height and weight parameters respectively. $\left\{ A_{H}, B_{H}, C_{H},D_{H} \right\}$ and $\left\{ A_{W}, B_{W}, C_{W},D_{W} \right\}$ the height and weight population parameters.

Thus, individual BMI trajectories were fitted for each child $i$ according to equation 3 where weight is expressed in kg and height in cm:

**Equation 3.**

$${BMI}_{i,j}=\frac{{Weight}_{i,j}}{\left( {{Height}_{i,j}}/{100} \right)^{2}}=\frac{\exp\left( A_{W_{i}} \right)+\exp\left( B_{W_{i}} \right)t_{i,j}+\exp\left( C_{W_{i}} \right)(1-\exp\left( -exp(D_{W_{i}}{) t}_{i,j}) \right)}{\left[ \left( \exp\left( A_{H_{i}} \right)+\exp\left( B_{H_{i}} \right)t_{i,j}+\exp\left( C_{H_{i}} \right)(1-\exp\left( -exp(D_{H_{i}}{) t}_{i,j}) \right) \right)/100 \right]^{2}}$$
