# Supplementary material for: A Novel Method to Describe Early Offspring Body Mass Index (BMI) Trajectories and to Study Its Determinants
Source: PLoS One. 2016 Jun 21;11(6):e0157766. doi: 10.1371/journal.pone.0157766 (PMC4915665; doi:10.1371/journal.pone.0157766)
Supplement: S2 File — (DOCX) [file pone.0157766.s002.docx]

**S2 File.** Models Adequacies


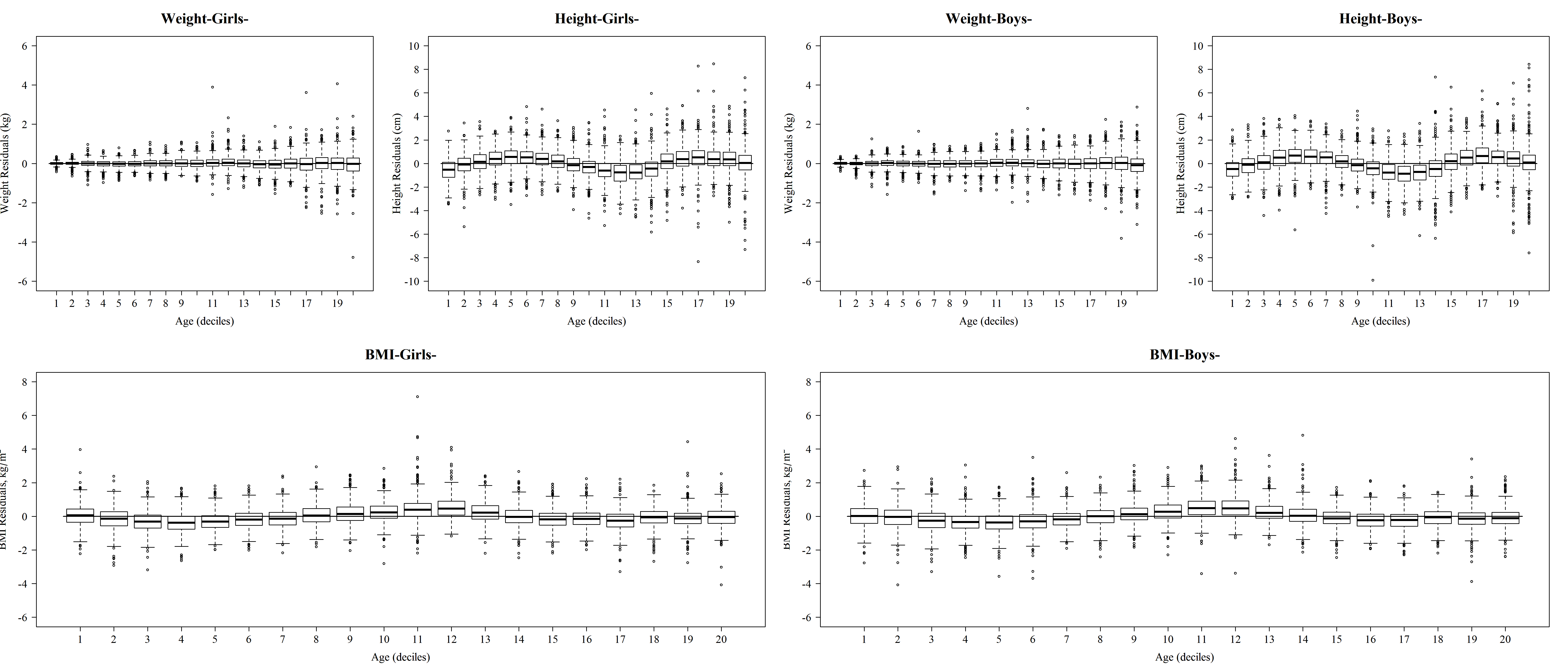


**Fig A.** Distribution over time of residuals of height and weight model and resulting difference between observed and calculated values of BMI by gender in the EDEN study (0-5 years)


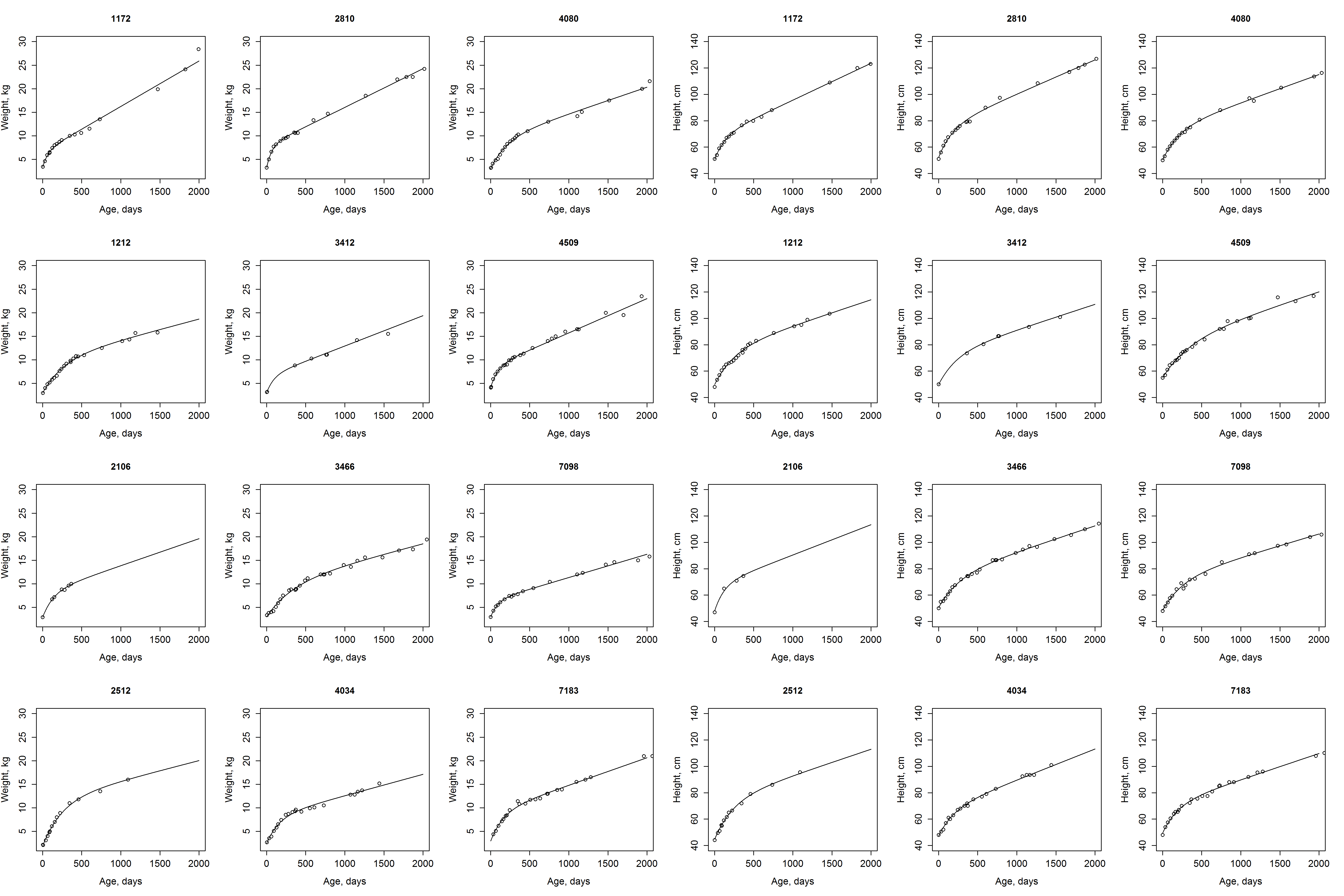


**Fig B.** Height/Weight measures (circles) and Fitted Trajectories (black lines) of Height (cm)/Weight (kg) for 12 randomly selected individuals from the EDEN study (0-5 years)

**
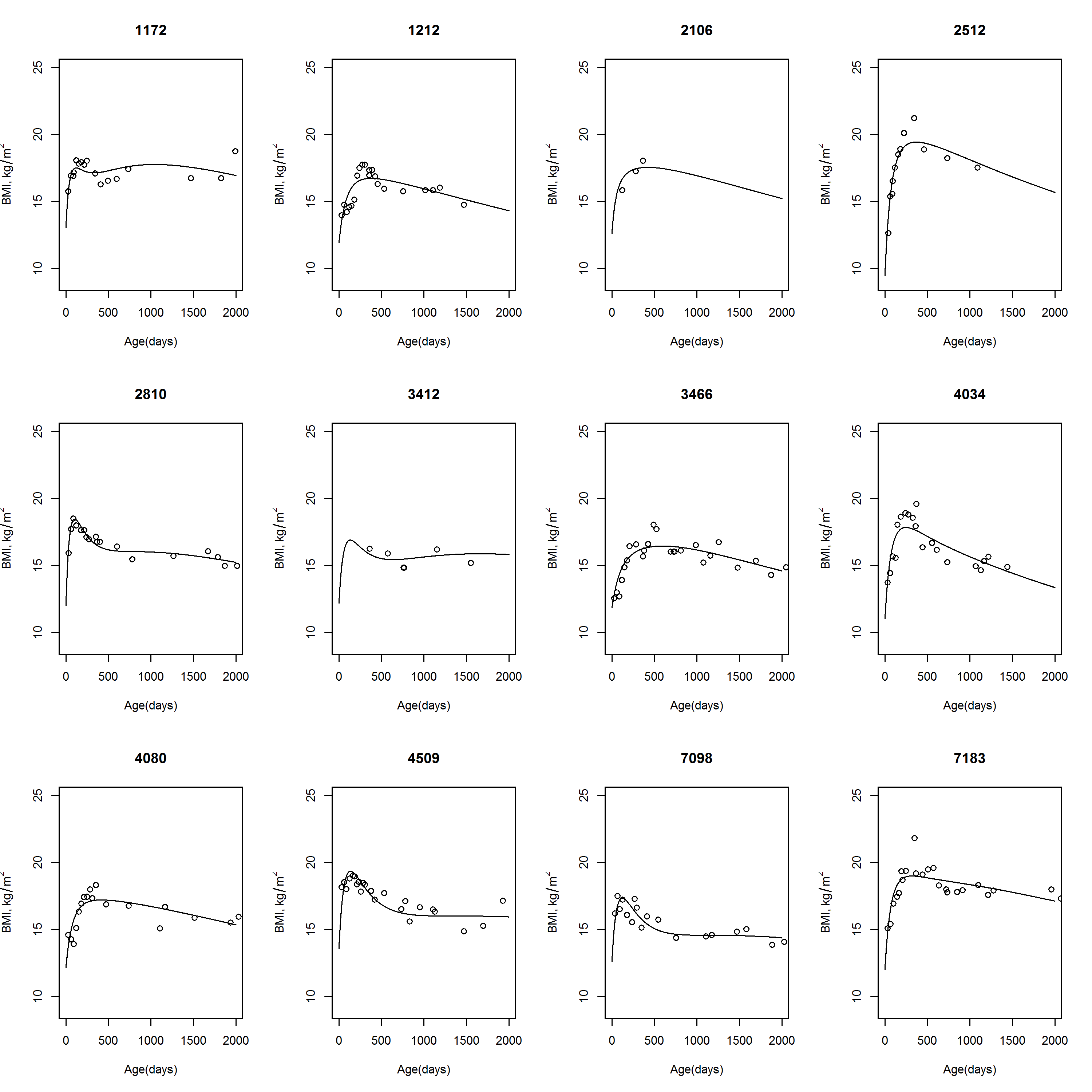
**

**Fig C.** BMI measures (circles) and Fitted Trajectories (black lines) of BMI (kg/m^2^) for 12 randomly selected individuals from the EDEN study (0-5 years)
