# Supplementary material for: A Novel Method to Describe Early Offspring Body Mass Index (BMI) Trajectories and to Study Its Determinants
Source: PLoS One. 2016 Jun 21;11(6):e0157766. doi: 10.1371/journal.pone.0157766 (PMC4915665; doi:10.1371/journal.pone.0157766)
Supplement: S3 File — (DOCX) [file pone.0157766.s003.docx]

**S3 File.** Dose Effect Analysis

**Table A.** Mean differences in Height and Weight Model Parameters for a 5 cigarettes per day increase in children whose mothers smoked throughout pregnancy, the EDEN study, France, 2003-2012

| Outcomes |  | A | |  | B | |  | C | |  | D | |
| --- | --- | --- | --- | --- | --- | --- | --- | --- | --- | --- | --- | --- |
|  |  | Birth length | |  | Growth Velocity | |  | Spurt of Growth | |  | Curvature Degree | |
|  |  | Extrapolation of Birth weight | |  | -Childhood- | |  | -First Months- | |  | -First Months- | |
|  |  | β_A_ | CI 95% |  | β_B_ | CI 95% |  | β_C_ | CI 95% |  | β_D_ | CI 95% |
| Height |  | -0.009* | [-0.013,-0.005] |  | 0.009 | [-0.006,0.025] |  | -0.001 | [-0.019,0.017] |  | 0.027 | [-0.005,0.059] |
| Weight |  | -0.026* | [-0.042,-0.011] |  | 0.018 | [-0.005,0.042] |  | 0.008 | [-0.017,0.034] |  | 0.014 | [-0.025,0.052] |
|  |  |  |  |  |  |  |  |  |  |  |  |  |
| Abbreviations: BMI, body mass index, CI, confidence interval. | | | | | | | | | | | | |
| ^a^Adjusted for maternal education level, BMI and age at delivery; breast-feeding duration; recruitment center; child's gender. | | | | | | | | | | | | |
| *P<0.05. | | | | | | | | | | | | |

**Table B.** Mean differences in Predicted Anthropometric Outcomes from Birth to 5 Years for a 5 cigarettes/day increase of the consumption of tobacco during the second and third trimesters of pregnancy^a^ (Two-steps method)

| Outcomes |  | Birth | |  | 3 months | |  | 6 months | |  | 1 year | |  | 2 years | |  | 4 years | |  | 5 years | |
| --- | --- | --- | --- | --- | --- | --- | --- | --- | --- | --- | --- | --- | --- | --- | --- | --- | --- | --- | --- | --- | --- |
|  |  | β | 95% CI |  | β | 95% CI |  | β | 95% CI |  | β | 95% CI |  | β | 95% CI |  | β | 95% CI |  | β | 95% CI |
| Length/Height, cm |  | -0.39* | [-0.58,-0.20] |  | -0.26* | [-0.44,-0.08] |  | -0.21* | [-0.40,-0.02] |  | -0.19 | [-0.39,0.01] |  | -0.21 | [-0.46,0.03] |  | -0.19 | [-0.51,0.13] |  | -0.16 | [-0.52,0.19] |
| Weight, g |  | -82* | [-125,-39] |  | -28 | [-83,27] |  | -2 | [-67,64] |  | 37 | [-45,118] |  | 86 | [-19,191] |  | 155 | [-1,312] |  | 188 | [0,376] |
| BMI, kg/m^2^ |  | -0.12 | [-0.25,0.01] |  | 0.06 | [-0.04,0.17] |  | 0.10 | [-0.01,0.21] |  | 0.15* | [0.04,0.26] |  | 0.19* | [0.09,0.30] |  | 0.20* | [0.10,0.30] |  | 0.19* | [0.09,0.30] |
| Height velocities, cm/month |  |  |  |  | 0.028* | [0.005,0.050] |  | 0.010 | [-0.004,0.024] |  | -0.001 | [-0.013,0.010] |  | -0.001 | [-0.007,0.005] |  | 0.002 | [-0.004,0.007] |  | 0.002 | [-0.003,0.008] |
| Weight velocities, kg/month |  |  |  |  | 0.010* | [0.001,0.019] |  | 0.008* | [0.001,0.015] |  | 0.005* | [0.001,0.010] |  | 0.003* | [0.000,0.006] |  | 0.003 | [-0.001,0.006] |  | 0.003 | [-0.001,0.006] |
| BMI velocities, kg/m^2^/month |  |  |  |  | 0.024 | [-0.008,0.057] |  | 0.010 | [-0.001,0.021] |  | 0.006* | [0.001,0.012] |  | 0.002 | [-0.001,0.005] |  | 0.000 | [-0.002,0.002] |  | -0.001 | [-0.002,0.001] |
| Age at BMI peak^b^, days |  |  |  |  |  |  |  | 6.79 | [-2.57,16.14] |  |  |  |  |  |  |  |  |  |  |  |  |
| BMI at BMI peak^b^, kg/m^2^ |  |  |  |  |  |  |  | 0.12* | [0.00,0.23] |  |  |  |  |  |  |  |  |  |  |  |  |
|  |  |  |  |  |  |  |  |  |  |  |  |  |  |  |  |  |  |  |  |  |  |
| Abbreviations: BMI, body mass index, CI, confidence interval. | | | | | | | | | | | | | | | | | | | | | |
| ^a^Adjusted for maternal education level, BMI and age at delivery; breast-feeding duration; recruitment center; child's gender. | | | | | | | | | | | | | | | | | | | | | |
| ^b^Analyses restricted to those with valid data for age and BMI at BMI peak (identifiable BMI peak and BMI peak before 732 days). The number of children available for analysis was 1490. | | | | | | | | | | | | | | | | | | | | | |
| **P*<0.05. | | | | | | | | | | | | | | | | | | | | | |
