# Supplementary material for: A Novel Method to Describe Early Offspring Body Mass Index (BMI) Trajectories and to Study Its Determinants
Source: PLoS One. 2016 Jun 21;11(6):e0157766. doi: 10.1371/journal.pone.0157766 (PMC4915665; doi:10.1371/journal.pone.0157766)
Supplement: S4 File — (DOCX) [file pone.0157766.s004.docx]

**S4 File.** Sensitivity Analyses

**Table A.** Mean Differences in Predicted Anthropometric Outcomes from Birth to 5 Years in each Maternal Smoking Category and the Reference Category (No smoking)^a^ in full-term children

| Outcomes by maternal smoking status |  | Birth | |  | 3 months | |  | 6 months | |  | 1 year | |  | 2 years | |  | 4 years | |  | 5 years | |
| --- | --- | --- | --- | --- | --- | --- | --- | --- | --- | --- | --- | --- | --- | --- | --- | --- | --- | --- | --- | --- | --- |
|  |  | β | 95% CI |  | β | 95% CI |  | β | 95% CI |  | β | 95% CI |  | β | 95% CI |  | β | 95% CI |  | β | 95% CI |
| Length/Height, cm |  |  |  |  |  |  |  |  |  |  |  |  |  |  |  |  |  |  |  |  |  |
| Exclusively the 1^st^ trimester |  | -0.43* | [-0.78,-0.08] |  | -0.05 | [-0.39,0.29] |  | 0.06 | [-0.31,0.43] |  | 0.06 | [-0.36,0.47] |  | -0.03 | [-0.54,0.48] |  | 0.08 | [-0.59,0.75] |  | 0.20 | [-0.56,0.96] |
| Late pregnancy |  | -0.69* | [-0.95,-0.42] |  | -0.48* | [-0.74,-0.23] |  | -0.35* | [-0.64,-0.07] |  | -0.26 | [-0.57,0.06] |  | -0.20 | [-0.58,0.19] |  | -0.07 | [-0.58,0.43] |  | -0.01 | [-0.58,0.57] |
| Weight, g |  |  |  |  |  |  |  |  |  |  |  |  |  |  |  |  |  |  |  |  |  |
| Exclusively the 1^st^ trimester |  | -17 | [-95,60] |  | -31 | [-139,76] |  | -8 | [-142,127] |  | 30 | [-141,200] |  | 53 | [-168,274] |  | 58 | [-274,391] |  | 59 | [-342,460] |
| Late pregnancy |  | -140* | [-199,-81] |  | -58 | [-139,24] |  | -17 | [-119,85] |  | 48 | [-81,177] |  | 140 | [-27,308] |  | 266* | [14,518] |  | 322* | [19,626] |
| BMI, kg/m^2^ |  |  |  |  |  |  |  |  |  |  |  |  |  |  |  |  |  |  |  |  |  |
| Exclusively the 1^st^ trimester |  | 0.15 | [-0.08,0.39] |  | -0.08 | [-0.30,0.14] |  | -0.06 | [-0.29,0.17] |  | 0.01 | [-0.21,0.23] |  | 0.07 | [-0.15,0.28] |  | 0.01 | [-0.20,0.23] |  | -0.03 | [-0.25,0.20] |
| Late pregnancy |  | -0.20* | [-0.38,-0.02] |  | 0.11 | [-0.06,0.28] |  | 0.15 | [-0.03,0.32] |  | 0.19* | [0.03,0.36] |  | 0.25* | [0.09,0.41] |  | 0.27* | [0.11,0.43] |  | 0.26* | [0.09,0.43] |
| Height velocities, cm/month |  |  |  |  |  |  |  |  |  |  |  |  |  |  |  |  |  |  |  |  |  |
| Exclusively the 1^st^ trimester |  |  |  |  | 0.061* | [0.015,0.106] |  | 0.017 | [-0.01,0.045] |  | -0.009 | [-0.032,0.015] |  | -0.003 | [-0.015,0.009] |  | 0.009 | [-0.002,0.021] |  | 0.011 | [-0.001,0.023] |
| Late pregnancy |  |  |  |  | 0.059* | [0.025,0.094] |  | 0.029* | [0.008,0.050] |  | 0.008 | [-0.01,0.026] |  | 0.005 | [-0.005,0.014] |  | 0.006 | [-0.003,0.014] |  | 0.005 | [-0.004,0.015] |
| Weight velocities, kg/month |  |  |  |  |  |  |  |  |  |  |  |  |  |  |  |  |  |  |  |  |  |
| Exclusively the 1^st^ trimester |  |  |  |  | 0.006 | [-0.012,0.025] |  | 0.008 | [-0.006,0.022] |  | 0.004 | [-0.005,0.013] |  | 0.001 | [-0.006,0.007] |  | 0.000 | [-0.007,0.007] |  | 0.000 | [-0.007,0.007] |
| Late pregnancy |  |  |  |  | 0.015* | [0.001,0.029] |  | 0.012* | [0.002,0.023] |  | 0.010* | [0.003,0.016] |  | 0.006* | [0.001,0.011] |  | 0.005 | [0.000,0.010] |  | 0.005 | [-0.001,0.010] |
| BMI velocities, kg/m^2^/month |  |  |  |  |  |  |  |  |  |  |  |  |  |  |  |  |  |  |  |  |  |
| Exclusively the 1^st^ trimester |  |  |  |  | -0.059 | [-0.127,0.008] |  | 0.011 | [-0.011,0.034] |  | 0.010 | [-0.001,0.022] |  | 0.001 | [-0.005,0.007] |  | -0.003 | [-0.007,0.001] |  | -0.003 | [-0.006,0.000] |
| Late pregnancy |  |  |  |  | 0.030 | [-0.021,0.081] |  | 0.009 | [-0.008,0.026] |  | 0.008 | [-0.001,0.016] |  | 0.003 | [-0.002,0.007] |  | 0.000 | [-0.003,0.003] |  | -0.001 | [-0.003,0.002] |
| Age at BMI peak^b^, days |  |  |  |  |  |  |  |  |  |  |  |  |  |  |  |  |  |  |  |  |  |
| Exclusively the 1^st^ trimester |  |  |  |  |  |  |  | 6.49 | [-12.82,25.79] |  |  |  |  |  |  |  |  |  |  |  |  |
| Late pregnancy |  |  |  |  |  |  |  | 4.22 | [-10.37,18.81] |  |  |  |  |  |  |  |  |  |  |  |  |
| BMI at BMI peak^b^, kg/m^2^ |  |  |  |  |  |  |  |  |  |  |  |  |  |  |  |  |  |  |  |  |  |
| Exclusively the 1^st^ trimester |  |  |  |  |  |  |  | -0.06 | [-0.30,0.17] |  |  |  |  |  |  |  |  |  |  |  |  |
| Late pregnancy |  |  |  |  |  |  |  | 0.15 | [-0.02,0.33] |  |  |  |  |  |  |  |  |  |  |  |  |
|  |  |  |  |  |  |  |  |  |  |  |  |  |  |  |  |  |  |  |  |  |  |
| Abbreviations: BMI, body mass index, CI, confidence interval. | | | | | | | | | | | | | | | | | | | | | |
| ^a^Adjusted for maternal education level, BMI and age at delivery; breast-feeding duration; recruitment center; child's gender. | | | | | | | | | | | | | | | | | | | | | |
| ^b^Analyses restricted to those with valid data for age and BMI at BMI peak (identifiable BMI peak and BMI peak before 732 days). The number of children available for analysis was 1532. | | | | | | | | | | | | | | | | | | | | | |
| *P*<0.05. | | | | | | | | | | | | | | | | | | | | | |

**Table B.** Mean Differences in Predicted Anthropometric Outcomes from 3 months to 5 Years in Postnatally exposed children versus non-exposed children^a^ (Two-steps method)

| Outcomes by maternal smoking status | | 3 months | |  | 6 months | |  | 1 year | |  | 2 years | |  | 4 years | |  | 5 years | |
| --- | --- | --- | --- | --- | --- | --- | --- | --- | --- | --- | --- | --- | --- | --- | --- | --- | --- | --- |
|  |  | β | 95% CI |  | β | 95% CI |  | β | 95% CI |  | β | 95% CI |  | β | 95% CI |  | β | 95% CI |
| Length/Height, cm | |  |  |  |  |  |  |  |  |  |  |  |  |  |  |  |  |  |
| Postnatal Smoking Exposure | | -0.07 | [-0.35,0.21] |  | -0.13 | [-0.42,0.16] |  | -0.18 | [-0.49,0.13] |  | -0.21 | [-0.58,0.17] |  | -0.25 | [-0.73,0.24] |  | -0.27 | [-0.82,0.28] |
| Weight, g |  |  |  |  |  |  |  |  |  |  |  |  |  |  |  |  |  |  |
| Postnatal Smoking Exposure | | -8 | [-92,77] |  | -23 | [-124,79] |  | -45 | [-170,80] |  | -56 | [-216,104] |  | -39 | [-274,197] |  | -26 | [-308,256] |
| BMI, kg/m^2^ |  |  |  |  |  |  |  |  |  |  |  |  |  |  |  |  |  |  |
| Postnatal Smoking Exposure | | 0.03 | [-0.14,0.19] |  | 0.02 | [-0.15,0.19] |  | 0.00 | [-0.16,0.17] |  | 0.00 | [-0.15,0.16] |  | 0.04 | [-0.11,0.18] |  | 0.05 | [-0.10,0.20] |
|  |  |  |  |  |  |  |  |  |  |  |  |  |  |  |  |  |  |  |
| Abbreviations: BMI, body mass index, CI, confidence interval. | | | | | | | | | | | | | | | | | | |
| ^a^Adjusted for maternal education level, BMI and age at delivery; breast-feeding duration; recruitment center; child's gender. | | | | | | | | | | | | | | | | | | |
| *P*<0.05. | | | | | | | | | | | | | | | | | | |

**Table C.** Mean Differences in Predicted BMI from 3 months to 5 Years in each Maternal Smoking Category and the Reference Category (No Smoking)^a^ (Two-steps method) using FP, Splines and IM in the EDEN study

| Outcomes by maternal smoking status and modeling method | | 3 months | | |  | 6 months | | |  | 1 year | | |  | 2 years | | |  | 4 years | | |  | 5 years | | |
| --- | --- | --- | --- | --- | --- | --- | --- | --- | --- | --- | --- | --- | --- | --- | --- | --- | --- | --- | --- | --- | --- | --- | --- | --- |
|  |  | β | 95% CI | SD |  | β | 95% CI | SD |  | β | 95% CI | SD |  | β | 95% CI | SD |  | β | 95% CI | SD |  | β | 95% CI | SD |
| BMI, kg/m^2^ |  |  |  |  |  |  |  |  |  |  |  |  |  |  |  |  |  |  |  |  |  |  |  |  |
| Exclusively the 1^st^ trimester | |  |  |  |  |  |  |  |  |  |  |  |  |  |  |  |  |  |  |  |  |  |  |  |
| Indirect Modeling | | -0.05 | [-0.27,0.17] | 0.11 |  | -0.04 | [-0.27,0.19] | 0.12 |  | 0.03 | [-0.19,0.26] | 0.11 |  | 0.10 | [-0.12,0.31] | 0.11 |  | 0.04 | [-0.17,0.25] | 0.11 |  | 0.00 | [-0.22,0.22] | 0.11 |
| Fractional Polynomials | | -0.03 | [-0.23,0.17] | 0.10 |  | 0.01 | [-0.20,0.22] | 0.11 |  | 0.04 | [-0.17,0.25] | 0.11 |  | 0.05 | [-0.15,0.24] | 0.10 |  | 0.05 | [-0.14,0.24] | 0.10 |  | 0.04 | [-0.15,0.24] | 0.10 |
| Splines | | -0.04 | [-0.24,0.15] | 0.10 |  | 0.03 | [-0.20,0.25] | 0.11 |  | 0.07 | [-0.16,0.29] | 0.12 |  | 0.06 | [-0.12,0.25] | 0.10 |  | 0.07 | [-0.11,0.26] | 0.10 |  | 0.09 | [-0.11,0.28] | 0.10 |
| Late pregnancy | |  |  |  |  |  |  |  |  |  |  |  |  |  |  |  |  |  |  |  |  |  |  |  |
| Indirect Modeling | | 0.08 | [-0.09,0.24] | 0.09 |  | 0.11 | [-0.07,0.28] | 0.09 |  | 0.16 | [-0.01,0.33] | 0.09 |  | 0.23* | [0.07,0.39] | 0.08 |  | 0.25* | [0.09,0.41] | 0.08 |  | 0.24* | [0.07,0.41] | 0.08 |
| Fractional Polynomials | | 0.08 | [-0.07,0.23] | 0.08 |  | 0.11 | [-0.05,0.27] | 0.08 |  | 0.14 | [-0.02,0.30] | 0.08 |  | 0.18* | [0.03,0.32] | 0.07 |  | 0.21* | [0.07,0.35] | 0.07 |  | 0.22* | [0.07,0.37] | 0.07 |
| Splines | | 0.07 | [-0.08,0.21] | 0.07 |  | 0.14 | [-0.02,0.31] | 0.09 |  | 0.19* | [0.02,0.35] | 0.09 |  | 0.19* | [0.05,0.33] | 0.07 |  | 0.22* | [0.08,0.36] | 0.07 |  | 0.24* | [0.10,0.39] | 0.07 |
|  |  |  |  |  |  |  |  |  |  |  |  |  |  |  |  |  |  |  |  |  |  |  |  |  |
| Abbreviations: BMI, Body Mass Index; CI, confidence interval; SD, Standard Deviation | | | | | | | | | | | | | | | | | | | | | | | | |
| ^a^Adjusted for maternal education level, BMI and age at delivery; breast-feeding duration; recruitment center; child's gender | | | | | | | | | | | | | | | | | | | | | | | | |
| **P*<0.05 | | | | | | | | | | | | | | | | | | | | | | | | |

**Table D.** Mean Differences in Height, weight and BMI obtained from clinical visits (1, 3 and 5 years) and by modeling in each Maternal Smoking Category and the Reference Category (No Smoking)^a^ (Two-steps method)

| Outcomes by maternal smoking status and by types of data | 1 year | |  | 3 years | | |  | | 5 years | | |
| --- | --- | --- | --- | --- | --- | --- | --- | --- | --- | --- | --- |
|  | β (n) | 95% CI |  | β (n) | 95% CI | |  | | β (n) | 95% CI | |
| Length/Height, cm |  |  |  |  |  | |  | |  |  | |
| Exclusively the 1^st^ trimester |  |  |  |  |  | |  | |  |  | |
| Data from Clinical visits | 0.04 (119) | [-0.43,0.51] |  | 0.08 (99) | [-0.66,0.81] | |  | | -0.15 (82) | [-1.19,0.89] | |
| Model-predicted Data (subsample with clinical visits data) | 0.08 (119) | [-0.35,0.52] |  | 0.06 (99) | [-0.62,0.74] | |  | | -0.15 (82) | [-1.11,0.81] | |
| Model-predicted Data (all children included in the main analyses) | 0.18 | [-0.24,0.60] |  | 0.09 | [-0.50,0.67] | |  | | 0.35 | [-0.40,1.11] | |
| Late pregnancy |  |  |  |  |  | |  | |  |  | |
| Data from Clinical visits | -0.14 (228) | [-0.50,0.22] |  | -0.17 (159) | [-0.78,0.44] | |  | | -0.25 (142) | [-1.09,0.58] | |
| Model-predicted Data (subsample with clinical visits data) | -0.24 (228) | [-0.55,0.08] |  | -0.17 (159) | [-0.74,0.39] | |  | | -0.26 (142) | [-1.03,0.51] | |
| Model-predicted Data (all children included in the main analyses) | -0.23 | [-0.55,0.08] |  | -0.13 | [-0.57,0.31] | |  | | 0.02 | [-0.55,0.58] | |
| Weight, g |  |  |  |  |  | |  | |  |  | |
| Exclusively the 1^st^ trimester |  |  |  |  |  | |  | |  |  | |
| Data from Clinical visits | 5 (118) | [-194,204] |  | -62 (99) | [-396,272] | |  | | -21 (82) | [-612,571] | |
| Model-predicted Data (subsample with clinical visits data) | 29 (118) | [-151,208] |  | 72 (99) | [-242,387] | |  | | -20 (82) | [-537,497] | |
| Model-predicted Data (all children included in the main analyses) | 75 | [-95,245] |  | 113 | [-158,383] | |  | | 137 | [-261,535] | |
| Late pregnancy |  |  |  |  |  | |  | |  |  | |
| Data from Clinical visits | 79 (228) | [-74,232] |  | 125 (160) | [-152,402] | |  | | 358 (141) | [-117,833] | |
| Model-predicted Data (subsample with clinical visits data) | 71 (228) | [-67,210] |  | 151 (160) | [-110,413] |  | | 324 (141) | | [-90,739] |  |
| Model-predicted Data (all children included in the main analyses) | 33 | [-95,161] |  | 189 | [-14,392] | |  | | 307* | [8,606] | |
| BMI, kg/m^2^ |  |  |  |  |  | |  | |  |  | |
| Exclusively the 1^st^ trimester |  |  |  |  |  | |  | |  |  | |
| Data from Clinical visits | -0.01 (118) | [-0.29,0.27] |  | -0.09 (98) | [-0.33,0.15] | |  | | 0.01 (82) | [-0.29,0.30] | |
| Model-predicted Data (subsample with clinical visits data) | 0.01 (118) | [-0.23,0.24] |  | 0.05 (98) | [-0.19,0.28] | |  | | 0.01 (82) | [-0.27,0.29] | |
| Model-predicted Data (all children included in the main analyses) | 0.03 | [-0.19,0.26] |  | 0.08 | [-0.13,0.28] | |  | | 0.00 | [-0.22,0.22] | |
| Late pregnancy |  |  |  |  |  | |  | |  |  | |
| Data from Clinical visits | 0.2 (228) | [-0.01,0.42] |  | 0.18 (158) | [-0.02,0.37] | |  | | 0.36* (141) | [0.12,0.60] | |
| Model-predicted Data (subsample with clinical visits data) | 0.23* (228) | [0.05,0.41] |  | 0.21* (158) | [0.02,0.41] | |  | | 0.33* (141) | [0.11,0.55] | |
| Model-predicted Data (all children included in the main analyses) | 0.16 | [-0.01,0.33] |  | 0.25* | [0.09,0.40] | |  | | 0.24* | [0.07,0.41] | |
| ^a^Adjusted for maternal education level, BMI and age at delivery; breast-feeding duration; recruitment center; child's gender. | | | | | | | | | | | |
| Abbreviations: BMI, body mass index, CI, confidence interval. | | | | | | | | | | | |
| **P*<0.05. | | | | | | | | | | | |
