# Supplementary material for: A Novel Method to Describe Early Offspring Body Mass Index (BMI) Trajectories and to Study Its Determinants
Source: PLoS One. 2016 Jun 21;11(6):e0157766. doi: 10.1371/journal.pone.0157766 (PMC4915665; doi:10.1371/journal.pone.0157766)
Supplement: S1 Table — (DOCX) [file pone.0157766.s005.docx]

**S1 Table.** Number of Measurements available for Modeling Height and Weight growth of Children (0-5 years) from the EDEN study, France, 2003-2012

| Age range | |  | No. of Subjects^a^ | | |  | No. of Measures | | |  | No. of Measures per Subject^b^ | | |
| --- | --- | --- | --- | --- | --- | --- | --- | --- | --- | --- | --- | --- | --- |
|  |  |  | Boys | Girls | Total |  | Boys | Girls | Total |  | Boys | Girls | Total |
| Weight^c^ | <6 months |  | 864 | 801 | 1,665 |  | 5,198 | 4,864 | 10,062 |  | 6(1-9) | 6(1-9) | 6(1-9) |
|  | 6 months - 1 year |  | 814 | 754 | 1,568 |  | 3,263 | 2,987 | 6,250 |  | 4(1-9) | 4(1-8) | 4(1-9) |
|  | 1 - 2 years |  | 788 | 721 | 1,509 |  | 2,838 | 2,686 | 5,524 |  | 4(1-9) | 4(1-9) | 4(1-9) |
|  | 2 - 4 years |  | 750 | 694 | 1,444 |  | 3,055 | 2,820 | 5,875 |  | 4(1-9) | 4(1-10) | 4(1-10) |
|  | >= 4 years |  | 635 | 573 | 1,208 |  | 1,496 | 1,308 | 2,804 |  | 2(1-5) | 2(1-4) | 2(1-5) |
|  | Whole Study Period |  | 865 | 801 | 1,666 |  | 15,850 | 14,665 | 30,515 |  | 20(2-30) | 19(2-32) | 19(2-32) |
| Height | <6 months |  | 863 | 801 | 1,664 |  | 4,765 | 4,449 | 9,214 |  | 6(1-9) | 6(1-8) | 6(1-9) |
|  | 6 months - 1 year |  | 808 | 753 | 1,561 |  | 3,045 | 2,756 | 5,801 |  | 4(1-9) | 4(1-8) | 4(1-9) |
|  | 1 - 2 years |  | 784 | 715 | 1,499 |  | 2,697 | 2,555 | 5,252 |  | 3(1-9) | 4(1-9) | 4(1-9) |
|  | 2 - 4 years |  | 748 | 691 | 1,439 |  | 2,819 | 2,576 | 5,395 |  | 4(1-9) | 4(1-10) | 4(1-10) |
|  | >= 4 years |  | 633 | 570 | 1,203 |  | 1,459 | 1,260 | 2,719 |  | 2(1-5) | 2(1-4) | 2(1-5) |
|  | Whole Study Period |  | 865 | 801 | 1,666 |  | 14,785 | 13,596 | 28,381 |  | 18(2-30) | 18(2-31) | 18(2-31) |
|  |  |  |  |  |  |  |  |  |  |  |  |  |  |
| ^a^Subjects with More Than One Measure | | | | | | | | | | | | | |
| ^b^Median (min-max) | | | | | | | | | | | | | |
| ^c^Excluding Birth Weight and including the Minimal Weight Measure in the First Four Days | | | | | | | | | | | | | |
